# Pain persists in DAS28 rheumatoid arthritis remission but not in ACR/EULAR remission: a longitudinal observational study

**DOI:** 10.1186/ar3353

**Published:** 2011-06-08

**Authors:** Yvonne C Lee, Jing Cui, Bing Lu, Michelle L Frits, Christine K Iannaccone, Nancy A Shadick, Michael E Weinblatt, Daniel H Solomon

**Affiliations:** 1 Division of Rheumatology, Immunology and Allergy, Brigham and Women's Hospital, 75 Francis Street, PBB-B3, Boston, MA, USA

## Abstract

**Introduction:**

Disease remission has become a feasible goal for most rheumatoid arthritis (RA) patients; however, patient-reported symptoms, such as pain, may persist despite remission. We assessed the prevalence of pain in RA patients in remission according to the Disease Activity Score (DAS28-CRP4) and the American College of Rheumatology/European League Against Rheumatism (ACR/EULAR) remission criteria.

**Methods:**

Data were analyzed from RA patients in the Brigham Rheumatoid Arthritis Sequential Study with data at baseline and 1 year. DAS28 remission was defined as DAS28-CRP4 <2.6. The ACR/EULAR remission criteria included (a) one or more swollen joints, (b) one or more tender joints, (c) C-reactive protein ≤1 mg/dl, and (d) patient global assessment score ≤1. Pain severity was measured by using the pain score from the Multi-Dimensional Health Assessment Questionnaire (MDHAQ). The associations between baseline clinical predictors and MDHAQ pain at baseline and 1 year were assessed by using multivariable linear regression.

**Results:**

Among the 865 patients with data at baseline and 1 year, 157 (18.2%) met DAS28-CRP4 remission criteria at both time points. Thirty-seven (4.3%) met the ACR/EULAR remission criteria at baseline and 1 year. The prevalence of clinically significant pain (MDHAQ pain ≥4) at baseline ranged from 11.9% among patients meeting DAS28-CRP4 remission criteria to none among patients meeting ACR/EULAR remission criteria. Patient global assessment, MDHAQ function, MDHAQ fatigue, MDHAQ sleep, and arthritis self-efficacy were significantly associated with MDHAQ pain in cross-sectional (*P *≤ 0.0005) and longitudinal analyses (*P *≤ 0.03). Low swollen-joint counts were associated with high MDHAQ pain in longitudinal analyses (*P *= 0.02) but not cross-sectional analyses. Other measures of inflammatory disease activity and joint damage were not significantly associated with MDHAQ pain at baseline or at 1 year.

**Conclusions:**

Clinically significant pain continues among a substantial proportion of patients in DAS28 remission but not among those in ACR/EULAR remission. Among patients in DAS28 remission, patient global assessment, disability, fatigue, sleep problems, and self-efficacy are strongly associated with pain severity at baseline and 1 year, whereas inflammatory disease activity and joint damage are not significantly associated with elevated pain severity at either baseline or 1 year.

## Introduction

Rheumatoid arthritis (RA) is a chronic inflammatory disease that causes significant pain and disability. Sixty-eight percent of RA patients rate pain as their highest priority for improvement, and 90% of RA patients rate pain as one of their top three priorities [1]. With the earlier initiation of disease-modifying therapies and the development of targeted immunomodulating agents [2], an increasing number of RA patients are able to achieve minimal disease activity, and some are able to achieve complete disease remission [3-5]. However, on a population level, mean pain ratings have remained the same over the past 20-year period [6], and 82% of RA patients who consider their disease to be "somewhat to completely controlled" continue to report moderate to severe pain levels [7].

In a study of Disease Activity Score (DAS28) remission in the Arthritis and Rheumatology Clinic of Kansas and the Rheumatoid Arthritis Evaluation Study databases, mean and median pain scores were 2.43 and 1.50 on a 0-to-10 visual analogue pain scale [8]. Although these values were relatively low, they were higher than the cut-point of 1.25, which separated patients who were satisfied with their health from those who were not. This observation indicated that, even among patients in DAS28 remission, many still have enough pain to negatively affect health satisfaction. It was not clear whether this pain was due to residual inflammatory disease activity or whether this pain was not inflammatory.

Presented with a patient with tender joints and/or pain but no other evidence for inflammatory disease, physicians must decide whether to escalate or add other disease-modifying therapy. If they decide not to increase immunomodulation, they are left with the dilemma of how to address the pain. Currently, very little data exist to guide this treatment decision. The American College of Rheumatology (ACR)/European League Against Rheumatism (EULAR) consensus conference for the definition of remission recognized the potential impact of non-inflammatory causes of pain and called for studies to investigate the role of non-RA pain among patients in remission [9].

In this article, we examine the prevalence of clinically significant pain among RA patients in remission according to the DAS28-CRP4 and the ACR/EULAR criteria. Among patients who met the DAS28-CRP4 criterion for remission, we also assess whether pain severity is associated with inflammation or other factors, such as fatigue and sleep problems, which may be indicative of a non-inflammatory chronic widespread pain syndrome.

## Materials and methods

### Study population

The primary cohort includes the 157 RA patients in the Brigham and Women's Hospital Rheumatoid Arthritis Sequential Study (BRASS) who met DAS28-CRP4 remission criteria [10,11] at both study entry and 1-year follow-up. BRASS is an observational, hospital-based cohort of 1,118 individuals with established RA. Inclusion criteria include age 18 years or older and a diagnosis of RA made by a board-certified rheumatologist. Additional details can be found in a past publication [12]. The Partners Institutional Review Board approved the study, and written informed consent was obtained from all participants.

### Remission definitions

The DAS28-CRP4 was calculated from the tender-joint count, swollen-joint count, C-reactive protein (CRP), and patient global assessment [13]. DAS28-CRP4 remission was defined as a score <2.6 [10]. To satisfy the ACR/EULAR remission criteria, participants had to have: 1) ≤1 swollen joint, 2) ≤1 tender joint, 3) CRP ≤1 mg/dl, and 4) patient global assessment score ≤1 (of 10) [14]. The patient global score was taken from the Multi-Dimensional Health Assessment Questionnaire (MDHAQ) item stating, "Considering all the ways that your illness affects you, rate how you are doing."

### Study procedures

Participants provided demographic and clinical information, including detailed lists of comorbidities and medication histories. A modified Charlson comorbidity index was constructed by using available data [15]. Hemiplegia and peripheral vascular disease were omitted from the index because these data were not collected. We also did not have data to distinguish between mild liver disease and moderate/severe liver disease, uncomplicated diabetes versus diabetes with end-organ damage, and metastatic versus nonmetastatic tumors. In these cases, point values were assigned based on the less-severe condition (for example, all cases of liver disease were assigned 1 point; all cases of diabetes were assigned 1 point; all tumors were assigned 2 points).

Samples of blood were collected for a standard laboratory panel, including the CRP. Baseline hand radiographs were obtained, and Sharp scores were calculated based on joint-space narrowing and erosions. A physician performed a 28-joint count and assessment of disease activity. Participants also completed the MDHAQ, Mental Health Index-5 (MHI5) and the Arthritis Self Efficacy Scale (ASES). The MDHAQ is a validated instrument that includes assessments of pain, fatigue, and function, scored on 0-to-10 scales (high scores indicate more pain, more fatigue, and poor function) [16]. Specifically, the MDHAQ asks, "How much pain have you had because of your illness in the past week?" and "How much of a problem has fatigue or tiredness been for you in the past week?" The MDHAQ also includes a question regarding sleep problems, scored on a 0-to-3 scale (high scores indicate more sleep disturbances). The MHI5 is a validated five-item assessment of mental health, scored on a 1-to-100 scale (high scores indicate good mental health). The ASES is scored on a 10-to-100 scale (high scores indicate more self-efficacy) [17].

### Statistical analyses

Means and standard deviations (SDs) were calculated for normally distributed variables. Median values and interquartile ranges (IQRs) were determined for variables that were not normally distributed. Continuous independent variables were categorized based on tertiles.

To examine the prevalence of pain, clinically significant pain was defined as an MDHAQ pain score ≥4. This threshold was determined based on literature reports requiring pain-severity levels ≥4 of 10 or 40 of 100 for inclusion in clinical trials of pain medications to treat painful conditions, including arthritis [18].

Multivariable associations between baseline clinical variables and baseline MDHAQ pain score were examined by using multivariable linear regression. All multivariable models were adjusted for age and gender. The same methods were used to examine the longitudinal relation between baseline clinical variables and 1-year pain severity, except, in the primary multivariable analyses, baseline MDHAQ pain score was also included as a covariate. Secondary analyses were also conducted, adjusting for age and gender but excluding baseline MDHAQ pain score as a covariate. All statistical analyses were performed by using the SAS 9.2 software package (SAS Institute, Cary, NC, USA).

## Results

At baseline, the prevalence of DAS28-CRP4 remission was 24.9%, and the prevalence of ACR/EULAR remission was 8.8% among the 1,118 individuals in the BRASS cohort. The prevalence of remission did not differ significantly between those with 1-year follow-up data (25.3% met DAS28-CRP4 criteria; 8.6% met ACR/EULAR criteria) and those who were lost to follow-up (17.1% met DAS28-CRP4 criteria; 13.0% met ACR/EULAR criteria).

**Figure 1:**
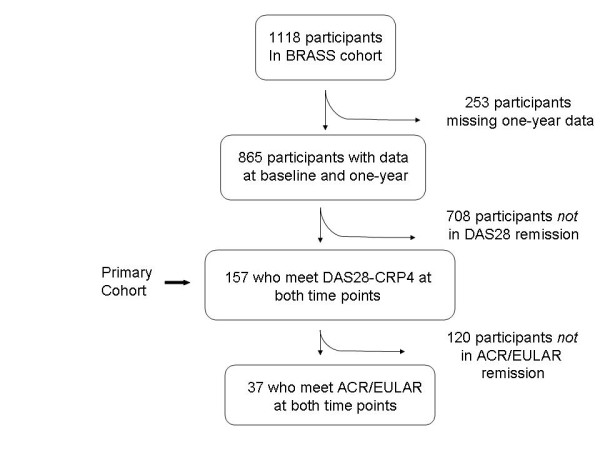
**Study disposition**. Flow diagram of the number of participants included in this study. ACR, American College of Rheumatology; BRASS, Brigham and Women's Hospital Rheumatoid Arthritis Sequential Study; DAS28, disease activity score in 28 joints; DAS28-CRP4, disease activity score in 28 joints calculated by using CRP and patient global assessment; EULAR, European League Against Rheumatism.

Among the 865 patients with both baseline and 1-year data, 157 (18.2%) patients remained in sustained DAS28-CRP4 remission at both baseline and 1 year (Figure 1). Thirty-seven (4.3%) also met ACR/EULAR criteria for remission at baseline and 1 year. All patients who met ACR/EULAR revised criteria for remission also met DAS28-CRP4 remission criteria.

### Baseline characteristics of patients in sustained remission

Among the 157 patients in sustained DAS28-CRP4 remission, mean age was 51.8 years; 135 (86.0%) were female patients (Table 1). Median disease duration was 6 years (IQR 2.0, 17.0), and 113 (72.4%) were seropositive. The median MHI5 score was 80.0 (IQR 70.0, 90.0) on a scale of 0 to 100, with 100 being perfect mental health. The median MDHAQ fatigue score was 2.5 (IQR 1.0, 5.0) on a scale of 0 to 10, with 10 being severe fatigue. Eighty-two participants (52.2%) were taking methotrexate; 71 (45.2%) were taking a biologic disease-modifying antirheumatic drug (DMARD), and 22 (14.0%) were taking a nonmethotrexate, nonbiologic DMARD. Thirty-six (22.9%) were taking a combination of methotrexate and a biologic DMARD. Four (2.6%) were taking a combination of methotrexate and a nonmethotrexate, nonbiologic DMARD, and seven (4.5%) were taking a combination of a biologic DMARD and a nonmethotrexate, nonbiologic DMARD. Eighty (51.0%) were taking nonsteroidal anti-inflammatory drugs, eight (5.1%) were taking opioid pain medications, and 16 (10.2%) were taking other pain medications (for example, tramadol and acetaminophen).

**Table 1:** Baseline characteristics of the 157 RA patients in sustained DAS28-CRP4 remission (at baseline and 1 year)

Clinical characteristics	Values
Demographic variables	
Age, years, mean (SD)	51.8 (14.0)
Female (%)	135 (86.0)
Caucasian (%)	145 (93.6)
Married/significant other (%)	112 (71.3)
Lives alone (%)	19 (14.1)
Ever smoker (%)	58 (39.2)
Body mass index, kg/m^2^, mean (SD)	25.0 (4.4)
Modified Charlson index (range, 0-23)	1.0 (0.0, 2.0)
	
Disease variables	
Disease duration, years	6.0 (2.0, 17.0)
Rheumatoid Factor/Anti-CCP positive (%)	113 (72.4)
Tender-joint count	0.0 (0.0, 0.0)
Swollen-joint count	0.0 (0.0, 2.0)
C-reactive protein, mg/L	1.2 (0.5, 2.7)
Patient global assessment (range, 0-10)	1.0 (0.0, 2.0)
DAS28-CRP4, mean (SD)	1.8 (0.4)
Total Sharp score (range, 0-448)	4.0 (0.0, 29.0)
Multi-Dimensional Health Assessment Function (range, 0-10)	0.3 (0.0-1.0)
Multi-Dimensional Health Assessment Pain (range, 0-10)	1.0 (0.5 2.5)
	
Psychiatric distress/Sleep variables	
Mental Health Index-5 (range, 0-100)	80.0 (70.0, 90.0)
Multi-Dimensional Health Assessment Sleep (range, 0-3)	1.0 (0.0, 1.0)
Multi-Dimensional Health Assessment Fatigue (range, 0-10)	2.5 (1.0, 5.0)
Arthritis Self-efficacy (range, 10-100)	82.5 (70.0, 90.0)
	
Medications	
Prednisone (%)	31 (19.8)
MTX (%)	82 (52.2)
bDMARD (%)	71 (45.2)
Non-MTX, non-bDMARD (%)	22 (14.0)
Combination MTX and bDMARD (%)	36 (22.9)
Combination MTX and non-MTX, non-bDMARD (%)	4 (2.6)
Combination bDMARD and non-MTX, non-bDMARD (%)	7 (4.5)
Nonsteroidal anti-inflammatory drugs (%)	80 (51.0)
Opioids (%)	8 (5.1)
Other pain medications (%)	16 (10.2)

anti-CCP, anti-cyclic citrullinated peptide; bDMARD, biologic DMARD; DAS28-CRP4, disease activity score in 28 joints calculated by using CRP and patient global assessment; MTX, methotrexate; RA, rheumatoid arthritis; SD, standard deviation. Continuous variables are listed as medians (interquartile ranges), and dichotomous variables are listed as numbers (%), unless otherwise noted.

### Pain severity at baseline and 1 year

**Figure 2:**
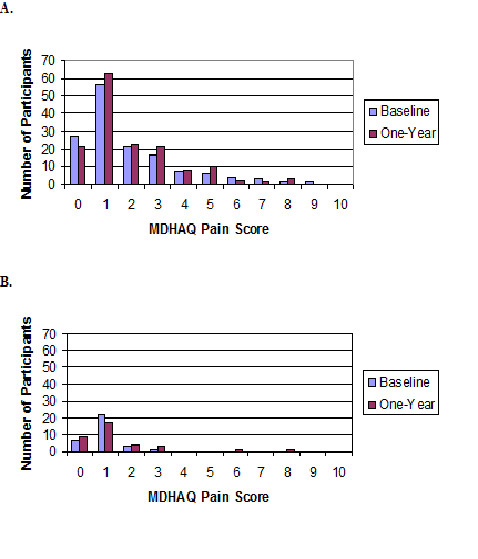
**Distribution of MDHAQ pain scores at baseline and 1 year**. The numbers depicted are among **(a) **RA patients in DAS28-CRP4 remission (*n *= 157), and **(b) **RA patients in ACR/EULAR remission (*n *= 37). ACR, American College of Rheumatology; DAS28-CRP4, disease activity score in 28 joints calculated by using CRP and patient global assessment; EULAR, European League Against Rheumatism; MDHAQ, Multi-Dimensional Health Assessment Questionnaire; RA, rheumatoid arthritis.

Among patients in sustained DAS28-CRP4 remission, the distribution of MDHAQ pain scores was skewed at baseline and 1 year, with the majority of patients having MDHAQ pain scores ≤2 (Figure 2). At baseline, 11.9% had MDHAQ pain scores ≥4, and 12.5% had MDHAQ pain scores ≥4 at 1 year. None of the 37 patients in sustained ACR remission had a MDHAQ pain rating ≥4 at baseline, and only two (5.7%) had MDHAQ pain ratings ≥4 at 1 year.

### Cross-sectional associations between baseline clinical variables and baseline pain

In multivariable analyses adjusted for age and gender, baseline patient global assessment ratings (*P *< 0.0001) and MDHAQ function scores (*P *< 0.0001) were the only disease-related variables significantly associated with high baseline MDHAQ pain scores (Table 2). Swollen-joint count, tender-joint count, and CRP were not significantly associated with MDHAQ pain scores. Sleep and psychosocial variables, including the baseline MDHAQ fatigue score (*P *< 0.0001) and the MDHAQ sleep-problems score (*P *= 0.0005) were strongly associated with high baseline MDHAQ pain scores. Lower ASES scores (*P *= 0.0005) and MHI scores (*P *= 0.004) were also significantly associated with high baseline MDHAQ pain scores.

**Table 2:** Cross-sectional associations between baseline clinical variables and baseline MDHAQ pain, adjusted for age and gender, among 157 RA patients in remission at baseline and 1 year

Variable	Mean MDHAQ Pain (95% CI)	*P*
Disease duration		0.44
≤3 years	1.4 (0.8, 2.0)	
4-11 years	1.8 (1.2, 2.4)	
≥12 years	1.7 (1.0, 2.4)	
Rheumatoid factor/cyclic citrullinated peptide status		0.59
Seropositive	1.5 (0.9, 2.1)	
Seronegative	1.7 (1.2, 2.2)	
Tender-joint count		0.79
0	1.6 (1.1, 2.1)	
1	1.5 (0.6, 2.4)	
≥2	1.8 (0.8, 2.9)	
Swollen-joint count		0.92
0	1.6 (1.1, 2.1)	
1	1.2 (0.0, 2.3)	
≥2	1.7 (1.1, 2.4)	
C-reactive protein		0.28
<0.7 mg/L	1.5 (0.9, 2.1)	
0.7-2.0 mg/L	1.5 (0.9, 2.1)	
≥2.1 mg/L	1.9 (1.3, 2.5)	
Patient global health assessment		**<0.0001**
<5	0.6 (0.1, 1.1)	
5-14	1.4 (0.8, 1.9)	
≥15	2.9 (2.4, 3.4)	
Sharp score		0.24
≤1	1.8 (1.1, 2.4)	
2-17	1.9 (1.2, 2.7)	
≥18	1.2 (0.5, 2.0)	
MDHAQ Function Scale		**<0.0001**
0	0.6 (0.1, 1.2)	
0.1-0.9	1.4 (0.9, 2.0)	
≥1.0	2.9 (2.3, 3.4)	
Mental Health Index 5		**0.004**
≤70	2.2 (1.6, 2.8)	
71-80	1.7 (1.1, 2.4)	
>80	1.1 (0.6, 1.7)	
MDHAQ Fatigue Scale		**<0.0001**
<1.0	0.9 (0.3, 1.4)	
1.0-3.9	1.2 (0.7, 1.8)	
≥4.0	2.9 (2.3, 3.4)	
MDHAQ Sleep Problems Score		**0.0005**
0	1.2 (0.7, 1.7)	
1	2.1 (1.5, 2.7)	
≥2	2.7 (1.6, 3.8)	
Arthritis Self-Efficacy Score		**0.0005**
<78	2.1 (1.6, 2.7)	
78-87	1.9 (1.3, 2.5)	
≥88	0.9 (0.3, 1.4)	
Modified Charlson Index		0.91
≤1	1.6 (1.2, 2.1)	
>1	1.6 (1.0, 2.2)	

MDHAQ, Multi-Dimensional Health Assessment Questionnaire; RA, rheumatoid arthritis.

*P *values from multivariable linear regression models.

### Longitudinal analyses examining associations between baseline clinical variables and pain at 1 year

In multivariable analyses adjusted for age, gender, and baseline MDHAQ pain score, baseline MDHAQ function score (*P *= 0.008), disease duration (*P *= 0.02), and patient global assessment (*P *= 0.03) were the only disease-related variables positively associated with MDHAQ pain scores at 1 year. Baseline swollen-joint count was negatively associated with MDHAQ pain scores at 1 year (*P *= 0.02). Baseline MDHAQ fatigue score (*P *= 0.0001), MDHAQ sleep-problems score (*P *= 0.001), and ASES score (*P *= 0.01) were all significantly associated with 1-year MDHAQ pain score (Table 3). In secondary analyses, excluding baseline MDHAQ pain score as a covariate, baseline patient global assessment, baseline MDHAQ function score, baseline MDHAQ fatigue score, baseline MDHAQ sleep-problems score and baseline ASES score all remained significantly associated with 1-year MDHAQ pain score (*P *< 0.0001).

**Table 3:** Longitudinal associations between baseline clinical variables and 1-year MDHAQ pain, adjusted for age, gender, and baseline MDHAQ pain, among 157 RA patients in remission at baseline and 1 year

Variable	Mean MDHAQ Pain (95% CI)	*P*
Disease duration		**0.02**
≤3 years	1.6 (1.1, 2.2)	
4-11 years	2.0 (1.6, 2.5)	
≥12 years	2.4 (1.8, 3.0)	
Rheumatoid factor/cyclic citrullinated peptide status		0.77
Seropositive	1.9 (1.5, 2.4)	
Seronegative	2.1 (1.5, 2.6)	
Tender-joint count		0.36
0	2.1 (1.6, 2.5)	
1	1.7 (1.0, 2.5)	
≥2	1.8 (0.9, 2.6)	
Swollen-joint count		**0.02**
0	2.2 (1.8, 2.6)	
1	1.3 (0.3, 2.3)	
≥2	1.5 (1.0, 2.1)	
C-reactive protein		0.77
<0.7 mg/L	1.9 (1.4, 2.5)	
0.7-2.0 mg/L	2.0 (1.5, 2.5)	
≥2.1 mg/L	2.0 (1.5, 2.5)	
Patient global health assessment		**0.03**
<5	1.6 (0.9, 2.2)	
5-14	1.7 (1.1, 2.2)	
≥15	2.5 (2.0, 3.0)	
Sharp score		0.53
≤1	2.1 (1.4, 2.7)	
2-17	2.2 (1.6, 2.9)	
≥18	1.8 (1.1, 2.5)	
MDHAQ Function Scale		**0.008**
0	1.6 (1.0, 2.2)	
0.1-0.9	1.5 (1.0, 2.0)	
≥1.0	2.7 (2.1, 3.2)	
Mental Health Index 5		0.43
≤70	2.2 (1.7, 2.4)	
71-80	1.7 (1.1, 2.3)	
>80	1.9 (1.4, 2.4)	
MDHAQ Fatigue Scale		**0.0001**
<1.0	1.2 (0.7, 1.8)	
1.0-3.9	1.8 (1.3, 2.3)	
≥4.0	2.8 (2.3, 3.3)	
MDHAQ Sleep Problems Score		**0.001**
0	1.6 (1.1, 2.0)	
1	2.3 (1.8, 2.8)	
≥2	3.1 (2.2, 4.0)	
Arthritis Self-Efficacy Score		**0.01**
<78	2.2 (1.7, 2.7)	
78-87	2.3 (1.8, 2.8)	
≥88	1.4 (0.9, 1.9)	
Modified Charlson Index		0.35
≤1	1.9 (1.5, 2.3)	
>1	2.2 (1.6, 2.7)	

MDHAQ, Multi-Dimensional Health Assessment Questionnaire; RA, rheumatoid arthritis. *P *values are from multivariable linear regression models.

## Discussion

Among patients in sustained DAS28-CRP4 remission for longer than 1 year, the prevalence of clinically significant pain was 11.9% at baseline and 12.5% at 1 year. No markers of inflammatory activity were associated with increased pain severity at baseline or 1 year. Patient global assessment, disability, fatigue, sleep problems, and self-efficacy were strongly associated with pain at both baseline and 1 year.

Although rheumatologists and patients typically think of RA remission as a painless state [19-21], our study shows that remission does not always equal a pain-free state. These findings are significant because pain is considered the most important patient-reported outcome in rheumatology [22], yet it persists in >10% of patients who are classified in remission by DAS28-CRP4 criteria.

The cause of pain in these patients may arise from inflammation, structural joint damage, and/or non-disease-related factors. Our data suggest that inflammation and structural joint damage are not major causes of pain, because CRP, swollen-joint count, tender-joint count, and Sharp scores were not significantly associated with increased pain severity at baseline or 1 year. However, patient global assessment and MDHAQ function scores were strongly associated with increased pain severity at baseline and 1 year. Disease duration was also significantly associated with pain severity at 1 year, although not at baseline.

The patient global assessment and MDHAQ function scores are often interpreted as measures of RA-related joint inflammation and damage, but these measures also reflect a wide range of non-inflammatory factors. Other studies have reported that the explained variance in patient global scores by inflammation is low (6.7%) [23], and MDHAQ function scores are elevated in a wide range of other conditions, including fibromyalgia, a non-inflammatory condition that is not associated with joint damage [24]. RA patients with fibromyalgia have significantly worse physical function compared with RA patients without fibromyalgia, even though sedimentation rates are only marginally higher [25]. Given these observations and the lack of association between Sharp scores, CRP, swollen-joint count, tender-joint count, and high MDHAQ pain scores, we believe that the associations between patient global assessment/MDHAQ function and MDHAQ pain may be attributed to non-RA-related factors, and that the likelihood that inflammation and joint damage are major contributors to pain is low.

The association between disease duration and 1-year MDHAQ pain may reflect joint damage that accrues with increasing disease duration, or it may reflect time-dependent changes in pain processing that lead to a hyperalgesic state. However, disease duration was not significantly associated with baseline MDHAQ pain. This finding weakens the argument that disease duration acts as a proxy for joint damage and raises the possibility that the association between disease duration and 1-year MDHAQ pain may be attributable to chance. Alternatively, these observations are compatible with the hypothesis that increased pain is due to a hyperalgesic state, such as fibromyalgia, that is established and/or enhanced between the baseline and 1-year visits.

The strong association between baseline fatigue, sleep problems, poor self-efficacy, and baseline and 1-year pain severity also supports the existence of an enhanced, non-inflammatory pain state among these patients with pain despite DAS28-CRP4 remission. Fatigue, sleep problems, and poor self-efficacy are part of a noninflammatory symptom cluster associated with "symptom intensification" syndromes [26-28]. These syndromes encompass conditions such as fibromyalgia, which manifest with a broad range of symptoms, including widespread pain, fatigue, sleep problems, and cognitive difficulties. The etiology of these syndromes is unclear but may be associated with deficits in central pain-processing mechanisms, such as central sensitization, that result in hyperalgesia and allodynia [26]. Thus, therapeutic strategies targeted toward correcting central pain mechanisms and improving sleep and self-efficacy may be more effective at decreasing pain than strategies focused on intensifying disease-modifying therapies.

Limitations of this study include the use of the DAS28-CRP4 threshold of 2.6 as the primary criterion for remission. Although the DAS28-CRP4 remission criterion is the most commonly used definition for remission, it is not a perfect measure of inflammatory disease activity. Critics have noted that the DAS28-CRP4 remission threshold of 2.6 permits patients to have multiple tender and swollen joints [29]. In this study, the maximum number of tender joints was three, and the maximum number of swollen joints was 10, confirming these assertions. However, neither tender-joint count nor swollen-joint count was significantly associated with pain severity in cross-sectional analyses. In longitudinal analyses, swollen-joint count was associated with low pain levels, rather than high pain levels. Together with the finding that CRP levels were not significantly associated with pain scores, these observations argue against inflammation as a cause of pain among patients in DAS28-CRP4 remission.

In recognition that the DAS28-CRP4 remission criterion may be too permissive of residual inflammation, we also examined the prevalence of clinically significant pain among patients meeting 2010 ACR/EULAR remission criteria. The prevalence of clinically significant pain in this group was much lower, none at baseline and 5.7% at 1 year. This low prevalence is likely due to the ACR/EULAR criterion limiting the patient global assessment score to ≤1. Because the patient global assessment is heavily influenced by pain [30], this criterion essentially excludes patients with high pain scores. It is not clear whether omitting these patients is appropriate because the ACR/EULAR remission criteria exclude patients with pain due to residual, undetected inflammation or is inappropriate because the ACR/EULAR criteria include patients with non-inflammatory causes of pain. Our data do not allow us to distinguish between these two possibilities because we do not have measurements of joint tenderness and swelling in the feet and ankles. We also do not have radiographic measures to assess subclinical inflammation. Future studies including detailed assessments of inflammation in the lower extremities and radiographic assessments of synovitis are needed to elucidate the relation between inflammation and pain severity among patients in DAS28-CRP4 remission.

An additional limitation of this study is the homogeneity of the study population. The study was conducted at a single academic center, and 93.6% of this population was Caucasian. Other studies have reported higher rates of pain among racial minorities [31], and associations between pain, sleep problems, fatigue, and self-efficacy may differ depending on race. Future studies are needed to evaluate these relationships among different populations.

## Conclusions

Pain continues to persist in a substantial subgroup of patients who meet the DAS28-CRP4 threshold for remission, whereas nearly all patients with clinically significant pain are excluded by the ACR/EULAR remission criteria. Among RA patients who meet the DAS28-CRP4 threshold for remission, pain was not associated with inflammatory markers and did not diminish despite continued control of inflammation over a 1-year period. Pain was associated with fatigue, sleep problems, and self-efficacy. These findings stress the importance of assessing alternative causes of pain and modifying therapy to address specific modulators of pain, rather than assuming that pain is an indicator of inflammation that should be treated by increasing immunosuppressive therapies.

## Abbreviations

ACR: American College of Rheumatology; ASES: Arthritis Self Efficacy Scale; BRASS: Brigham and Women's Hospital Rheumatoid Arthritis Sequential Study; CRP: C-reactive protein; DAS28: disease activity score in 28 joints; DAS28-CRP4: disease activity score in 28 joints calculated by using CRP and patient global assessment; DMARD: disease-modifying antirheumatic drug; EULAR: European League Against Rheumatism; IQR: interquartile range; MDHAQ: Multi-Dimensional Health Assessment Questionnaire; MHI5: Mental Health Index-5; RA: rheumatoid arthritis; SD: standard deviation.

## Competing interests

YCL has stock in Merck, Novartis, and Elan Corporation and has a research grant from Forest. NAS receives research grant support from Biogen Idec, Crescendo Biosciences, and Amgen. MEW receives support from Biogen Idec and Crescendo Biosciences. DHS receives research support from Abbott and Amgen. No other authors have competing interests.

## Authors' contributions

YCL conceived the hypothesis for the manuscript, participated in data collection, conducted the initial statistical analyses, wrote the first draft of the manuscript, and had primary responsibility for the manuscript process. JC, BL, and MLF contributed to data management and statistical analyses. CKI, NAS, and MEW participated in the design of the study and the interpretation of data. DHS participated in study design and analysis and interpretation of data. All authors critically reviewed, contributed to, and approved the final manuscript. All authors read and approved the final manuscript.
